# Longitudinal analysis of carotenoid content in preterm human milk

**DOI:** 10.1007/s00431-024-05485-8

**Published:** 2024-03-21

**Authors:** Adi Uretzky, Dror Mandel, Anat Schwartz, Kira Kaganov, Daphna Mezad-Koursh, Laurence Mangel, Ronit Lubetzky

**Affiliations:** 1 Department of Neonatology, Tel Aviv Medical Center, Dana Dwek Children’s Hospital, Tel Aviv, 6997801 Israel; 2 Department of Pediatrics, Tel Aviv Medical Center, Dana Dwek Children’s Hospital, Tel Aviv, 6997801 Israel; 3 Tel Aviv Medical Center, Division of Ophthalmology, Tel Aviv, 6997801 Israel; 4 Faculty of Medicine, Tel Aviv University, Tel Aviv, Israel

**Keywords:** Carotenoids, Human breast milk (HM), Lutein, Retinopathy of prematurity (ROP)

## Abstract

To describe the variability in carotenoid content of human milk (HM) in mothers of very to extremely low birth weight preterm infants throughout lactation and to explore the relationship between lutein in HM and the occurrence of retinopathy of prematurity (ROP) in preterm infants. We recruited healthy mothers along with their preterm infants that were born at gestational age 24 + 2 to 29 + 6 weeks or with a birth weight under 1500 g and were exclusively breastfed HM. Each participant provided up to 7 HM samples (2–10 ml) on day 0–3 and once a week until 6 weeks. Additionally, when possible, a blood sample was collected from the infant at week 6. Concentrations of the major carotenoids (lutein, zeaxanthin, beta-carotene, and lycopene) in all HM and blood samples were assessed and compared. Thirty-nine mother-infant dyads were included and 184 HM samples and 21 plasma samples were provided. Mean lutein, zeaxanthin, beta-carotene, and lycopene concentration decreased as lactation progressed, being at their highest in colostrum samples (156.9 vs. 66.9 vs. 363.9 vs. 426.8 ng/ml, respectively). Lycopene (41%) and beta-carotene (36%) were the predominant carotenoids in colostrum and up to 2 weeks post-delivery. Inversely, the proportion of lutein and zeaxanthin increased with lactation duration to account for 45% of the carotenoids in mature HM. Lutein accounted for 58% of the carotenoids in infant plasma and only 28% in HM. Lutein content of transition and mature HM did not differ between mothers of ROP and non-ROP infants.

*Conclusion* Carotenoid content of HM was dynamic and varied between mothers and as lactation progressed. Infant plasma displayed a distinct distribution of carotenoids from HM.

**Supplementary Information:**

The online version contains supplementary material available at 10.1007/s00431-024-05485-8.

## What is Known

•*Carotenoids have antioxidant properties and their contents is dynamic.*

•*Preterm infants are susceptible to oxidative damage, which is a key factor in the pathogenesis process of ROP.*

**Supplementary Information:**

The online version contains supplementary material available at 10.1007/s00431-024-05485-8.

## What is New

•*We provide a longitudinal analysis of carotenoid content in human milk of very and extremely low birth weight (VLBW and ELBW) preterm infants.*

•*Levels of carotenoid in human milk were similar in both mothers of ROP and non-ROP infants.*

## Introduction

Human milk (HM) has a dynamic composition and evolves throughout lactation to meet infant’s needs. Carotenoids cannot be synthesized by human and must therefore be provided with the diet. The major carotenoids in HM and human blood are lutein, beta-carotene, beta-cryptoxanthin, and lycopene [1–3]. It is known that carotenoids have antioxidant and anti-inflammatory properties [4]. Accumulating evidence has implicated carotenoids in the fetal development of the macula [5, 6] and in the developing infant brain [7]. After birth, lutein and other carotenoids are supplied to the infant by HM or alternatively by some infant formulas [1, 6]. Maternal dietary patterns affect the carotenoid content in HM [3, 8, 9]. Several studies have reported that dietary lutein intake led to a progressive increase in the lutein content of HM [10, 11]. Furthermore, carotenoid supplementation in infant formula has raised plasma carotenoid concentration [12]. Preterm infants may be at risk for carotenoid deficiency, as they were deprived of an essential placental supply that occurs during the last weeks of pregnancy and may also have poorer absorption and reduced retention of nutrients due to their immature organs. Carotenoid content was shown to be lower in preterm than in full-term colostrum except for lutein which remained unaffected by the gestation age of the infant [13].

Retinopathy of prematurity (ROP) is a retinal neovascular disorder that affects preterm infants and constitutes a major cause of visual impairment and blindness [14]. Oxidative damage by oxygen free radicals has been identified as the key factor in the pathogenesis process of ROP [15, 16]. Preterm infants are susceptible to oxidative damage may lead to an abnormal development of the vascular tissue [17]. Lutein is thought to have a protective role against oxidative and light damage [14, 18, 19]. However, the physiological mechanism through which HM may protect against the development of ROP is poorly understood. To the best of our knowledge, no study has investigated the carotenoid content in HM produced by lactating mothers of very/extremely low birth weight (VLBW and ELBW) preterm infants and the potential link between its lutein content and ROP.

We conducted this prospective case study to assess first the carotenoid profile of preterm HM in mothers throughout the first weeks of lactation and second the relationship between lutein levels in HM and the occurrence of ROP in their preterm infants.

## Materials and methods

### Study Design and participants

The study was approved by our local institutional review board (0262-17-TLV) and written informed consent was obtained from all participants. Healthy exclusively lactating mothers of preterm infants born at gestational age (GA) 24 + 2/7 to 29 + 6/7 weeks or with a birth weight (BW) under 1500 g were enrolled along with their preterm infants at the Dana Dwek Children’s hospital of the Tel Aviv Medical Center between May 2018 and August 2020. Criteria for exclusion were maternal diabetes mellitus, pregnancy induced hypertension, preterm infants with congenital malformations, or any retinal disease. Per hospital guidelines, all infants in this study were fed according to standardized neonatal intensive care unit (NICU) feeding protocol with an exclusive HM-based diet when possible and complementary infant formula when necessary. Participants were requested to complete a questionnaire to collect demographic and clinical data.

### Collection of milk and blood samples

HM sample collection was standardized for all subjects as follows to avoid the influence of circadian and within-feed variations on HM components. HM samples of 2–10 ml were collected by electric pumping following the full milk expression session of the day (between 13:00 and 17:00), at days 0–3 (Colostrum), and once a week (+/- 2 days) until 6 weeks of age. In addition, when possible, plasma was prepared from an aliquot of 1 ml of blood collected from the infant at week 6, during the NICU routine blood test.

All milk and plasma samples were stored frozen at -80 °C, covered with aluminum foil to minimize light exposure at the Tel Aviv Medical Center for periods up to 9 months (average 3.2 months), until shipment.

### Laboratory methods

#### Carotenoid analysis in HM samples

HM samples were transferred frozen to the laboratory of Frutarom Ltd. where they were further stored frozen at -80 °C until analysis. The contents of lutein, beta-carotene, zeaxanthin, and lycopene in HM samples were measured by gradient reversed-phase high-performance liquid chromatography with UV detection by a method modified from Chauveau-Duriot et al. [20] (See Supplementary Methods).

#### Carotenoid analysis in plasma samples

Carotenoid analysis in plasma samples was performed in the Department of Nutrition-Health and Lipid Biochemistry at the French Institute for Fats and Oils in Canéjan, France. The concentration of carotenoid (alpha and beta-carotene, lycopene, lutein, zeaxanthin) in plasma was determined by high-performance liquid chromatography (Thermo Scientific, Malboeuf, France) with diode array detector, according to the modified method of Chauveau-Duriot et al. [20] (See Supplementary Methods).

### Retinopathy of prematurity

We defined ROP according to the International Classification of ROP (ICROP of 2005) [21]. Premature infants were evaluated following our local NICU protocol in accordance with the American Academy of Pediatrics recommendations [22]. First evaluation will be at 31 weeks post-menstrual age for infants born at GA between 22 and 27 weeks and at four weeks after birth for infants born at GA > 28 weeks. Until the retina has matured completely, follow-up evaluations will be conducted at the ophthalmologist’s discretion.

### Statistical analysis

Continuous data were expressed as means ± standard deviations (SD) or median with interquartile (IQR), according to distribution. Concentrations of lutein, zeaxanthin, beta-carotene, and lycopene in HM and plasma, were expressed as means ± standard error of the mean (SEM) or median with interquartile range (IQR). Categorical parameters were expressed as frequencies and proportions (%). The normality was assessed by Shapiro-Wilk tests. The Mann-Whitney test was applied to preliminary analyze the differences in carotenoid content of transition and mature milk as a function of independent variables such as maternal diet, gestational diabetes mellitus (GDM), alcohol consumption during breastfeeding, parity, smoking status (use and exposure), mode of delivery, previous breastfeeding experience or vitamin supplementation and to compare continuous variables between ROP diagnosed infants and non-ROP infants. Pearson’s r test was applied to assess the relationship between body mass index (BMI), maternal age, and carotenoid content of HM. Because lutein and beta-carotene levels displayed irregular distribution, we used a logarithmic transformation that converted the distribution to normal for the analysis of variance (ANOVA) tests. We applied a three-way ANOVA to further evaluate the contribution of maternal diet, mode of delivery and vitamin supplementation during lactation to the variability in lutein and beta-carotene contents of HM. Colostrum samples were not included in the ANOVA analysis due to the limited number of samples. Spearman’s Rho correlation analysis assessed the relationship between carotenoid levels in infant plasma and those found in HM at week 6 post-delivery. Missing data have been handled pairwise for frequencies and correlation tests and listwise for Mann-Whitney and ANOVA tests. The IBM SPSS Statistics for Windows, version 29, was used to carry out the statistical analyses. All p-values were 2-sided and *p* < 0.05 was considered statistically significant.

## Results

**Fig. 1:**
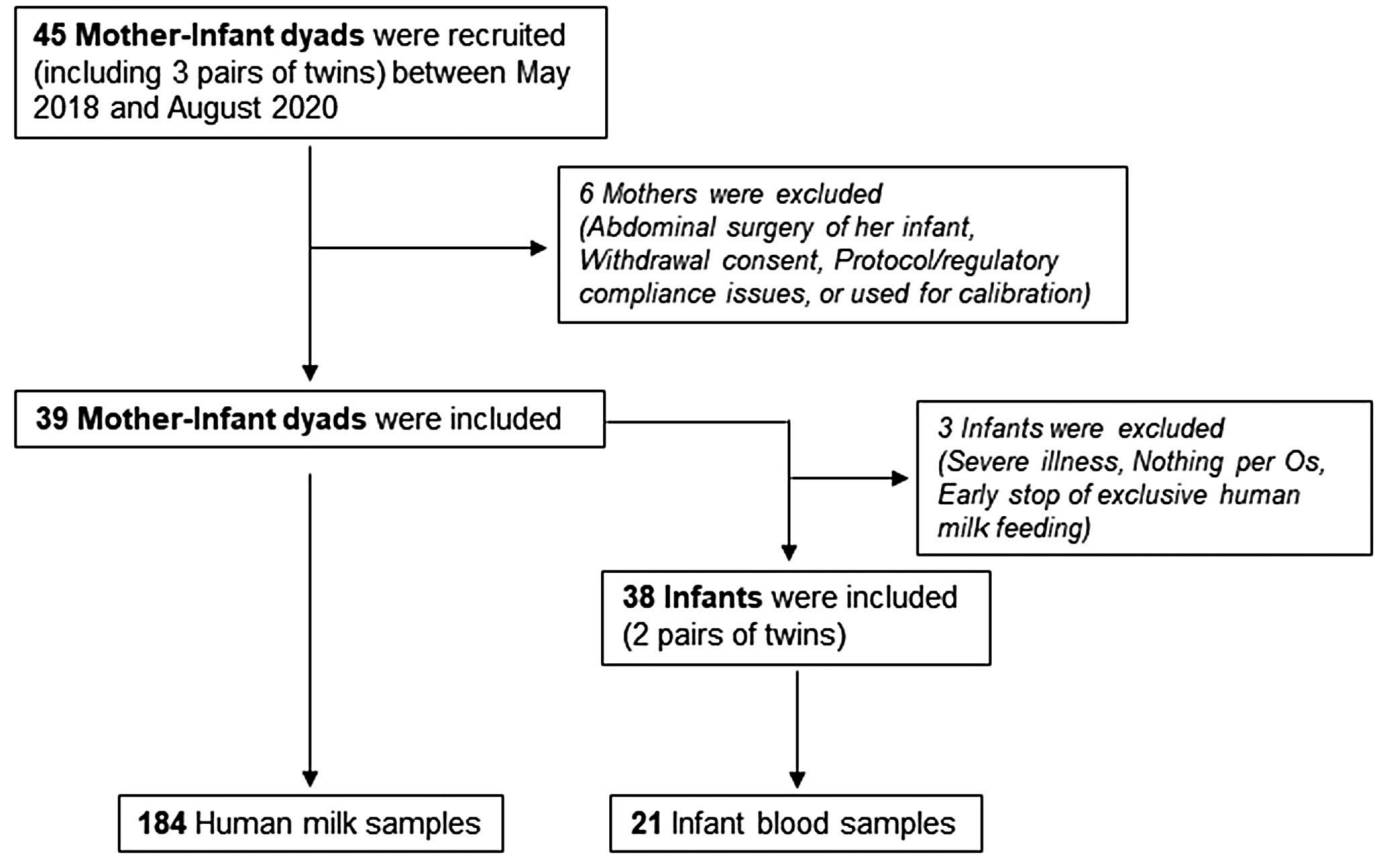
Flowchart of the study cohort

Forty-five mothers of preterm infants were recruited as depicted in Fig. 1. Three of them were mothers of twin infants. A total of 184 samples of HM were collected from 39 included mothers, and 56% of them provided five or more samples out of the seven required. The 38 included neonates provided a total of 21 blood samples. Collected maternal and neonatal data are presented in Table 1. Mothers were on average 32 years old and 10% of them were vegetarian. Twenty-three mothers (59%) took vitamin supplements while breastfeeding. Smoking or smoking exposure during breastfeeding was recorded in 18% of the mothers. The median (IQR) infant GA was 29 (27–30) weeks with a median BW of 1082.5 (940-1306.3) grams. The median (IQR) length of hospitalization stay of the neonates was 57.5 (40-90.5) days and seven (18%) of these infants developed ROP.

**Fig. 2:**
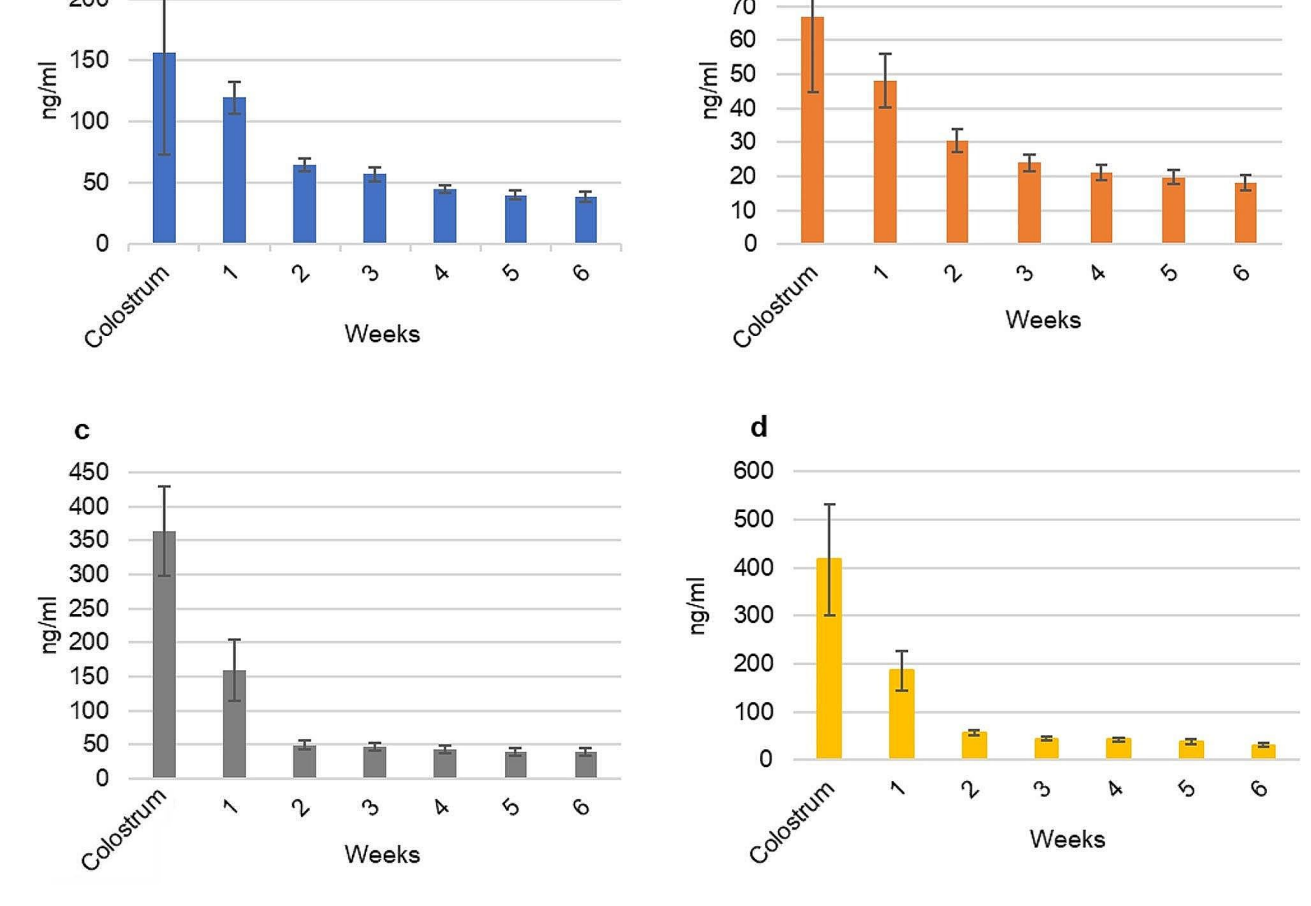
Concentrations (mean (± SEM)) of **a** lutein, **b** zeaxanthin, **c** beta-carotene and **d** lycopene throughout the first 6 weeks of lactation (colostrum and milk week (MW) 1 to 6)

The 184 expressed HM samples are distributed as four samples of colostrum and 27 and 31 samples of transition milk in the first and second week, respectively. Mature milk samples consisted of 33 samples in the third and fourth weeks, 29 samples in the firth week, and 27 samples in the last week of the HM collection period. Longitudinal lutein, zeaxanthin, beta-carotene, and lycopene concentrations during the first 6 weeks of lactation are presented in Fig. 2. All four carotenoids appeared to be at their maximum in colostrum and decreased as lactation progressed. Lutein concentration in colostrum was 4 times higher than in mature milk. On average (± SEM) lutein content of preterm HM decreased from 156.9 ± 84.1 ng/ml to 38.6 ± 4 ng/ml at week 6 post-delivery. A similar decreasing trend was observed for zeaxanthin (from 66.9 ± 22.3 to 18.1 ± 2.4 ng/ml), beta-carotene (from 363.9 ± 65.6 to 39.1 ± 6.4 ng/ml) and lycopene (from 426.8 ± 162.9 to 31.4 ± 3.9 ng/ml). In addition, HM displayed a great variability in its carotenoid content between mothers and there was an internal variation in the carotenoid distribution in each mother at the mature milk stage (Online Resource Fig. S1).

**Fig. 3:**
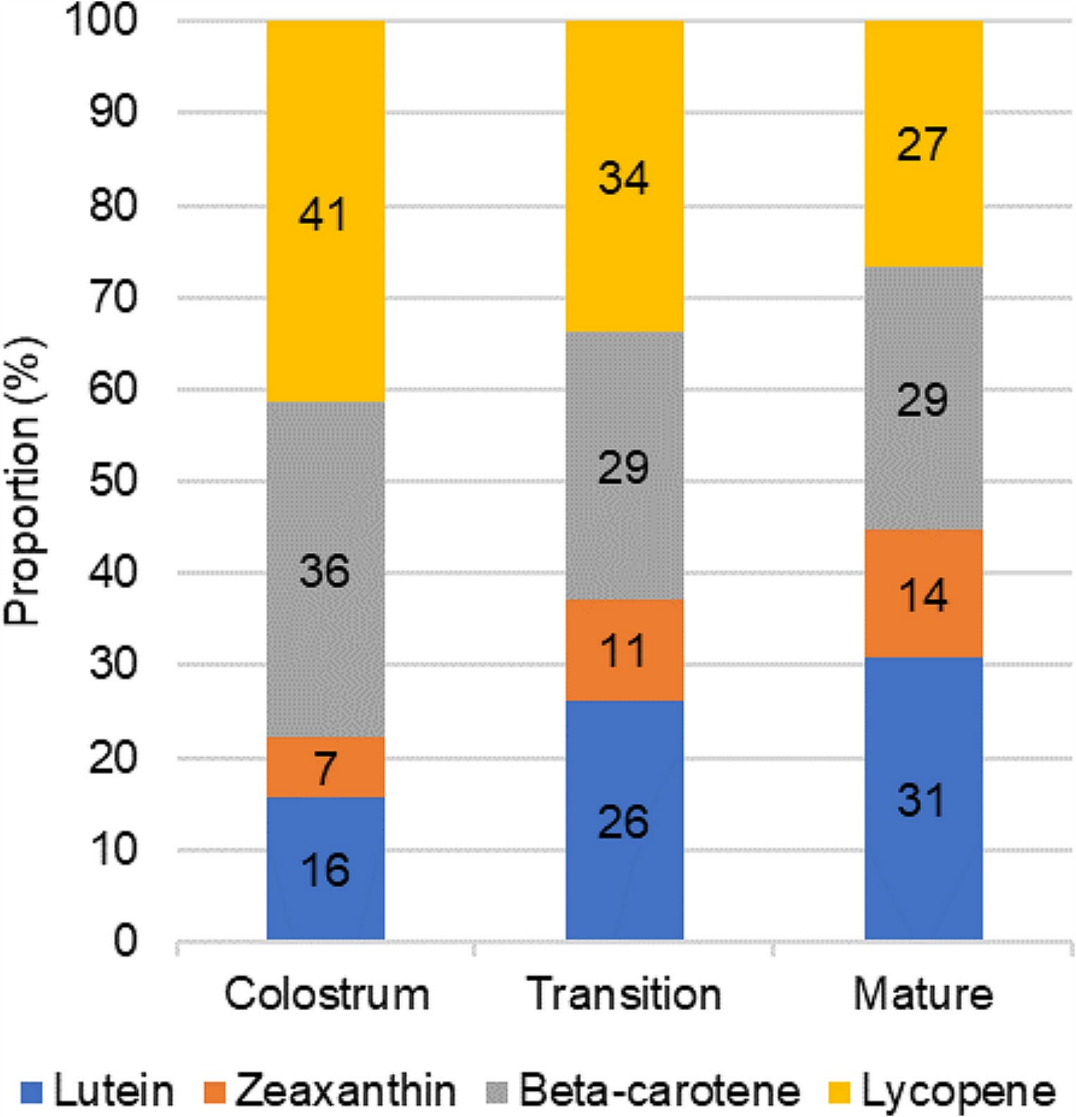
Proportions of carotenoids throughout lactation stages (colostrum, transition and mature milk)

Lycopene (41%) and beta-carotene (36%) were the predominant carotenoids at early stages of lactation (colostrum) and up to 2 weeks post-delivery (transition milk) to reach a lower proportion in mature milk. Inversely, lutein and zeaxanthin proportions increased with lactation to account for 45% of the carotenoids in mature HM (Fig. 3). Carotenoid content in transition and mature HM was affected by maternal diet, mode of delivery, and vitamin supplementation during breastfeeding but not by parity, alcohol consumption and smoking status during breastfeeding, previous breastfeeding experience, and GDM. Lutein and beta-carotene levels were significantly higher in vegetarian mothers than in omnivorous mothers and lower in mothers who had a cesarean delivery versus vaginal delivery at the transition and mature milk stages (Online Resource Table S1). Mothers who took vitamin supplementation during lactation had significantly higher median concentrations of beta-carotene than those who did not (81 versus 34.7 ng/ml, *p* = 0.049 and 46 versus 24.8 ng/ml, *p* = 0.041 in transition and mature milk, respectively). Maternal age and BMI did not correlate with the carotenoid content at the transition or mature milk stage. A three-way ANOVA was used to examine the main effects and interactions of maternal diet, mode of delivery and vitamin supplementation during lactation on lutein and beta-carotene levels of HM. The main effect for maternal diet yielded an F ratio of F(1, 27) = 8.5, *p* = 0.007, partial eta-squared (ηp^2^) = 0.240 and F(1, 33) = 8, *p* = 0.008, ηp^2^ = 0.195 for lutein in transition and mature milk respectively, such that lutein contents were significantly higher for vegetarian mothers, adjusted for the mode of delivery and vitamin supplementation. Similarly, vegetarian mothers had higher levels of beta-carotene in mature and transition milk when adjusted for mode of delivery and vitamin supplementation, (F(1, 27) = 15.8, *p* < 0.001, ηp^2^ = 0.370 and F(1, 33) = 11.3, *p* = 0.002, ηp^2^ = 0.256 respectively). The interaction effect of all three independent variables was significant only for the beta-carotene content and only in transition milk (F(1, 27) = 9.6, *p* = 0.004), however the measure of this effect size was smaller (ηp^2^ = 0.263) than for the maternal diet alone (ηp^2^ = 0.370).

**Fig. 4:**
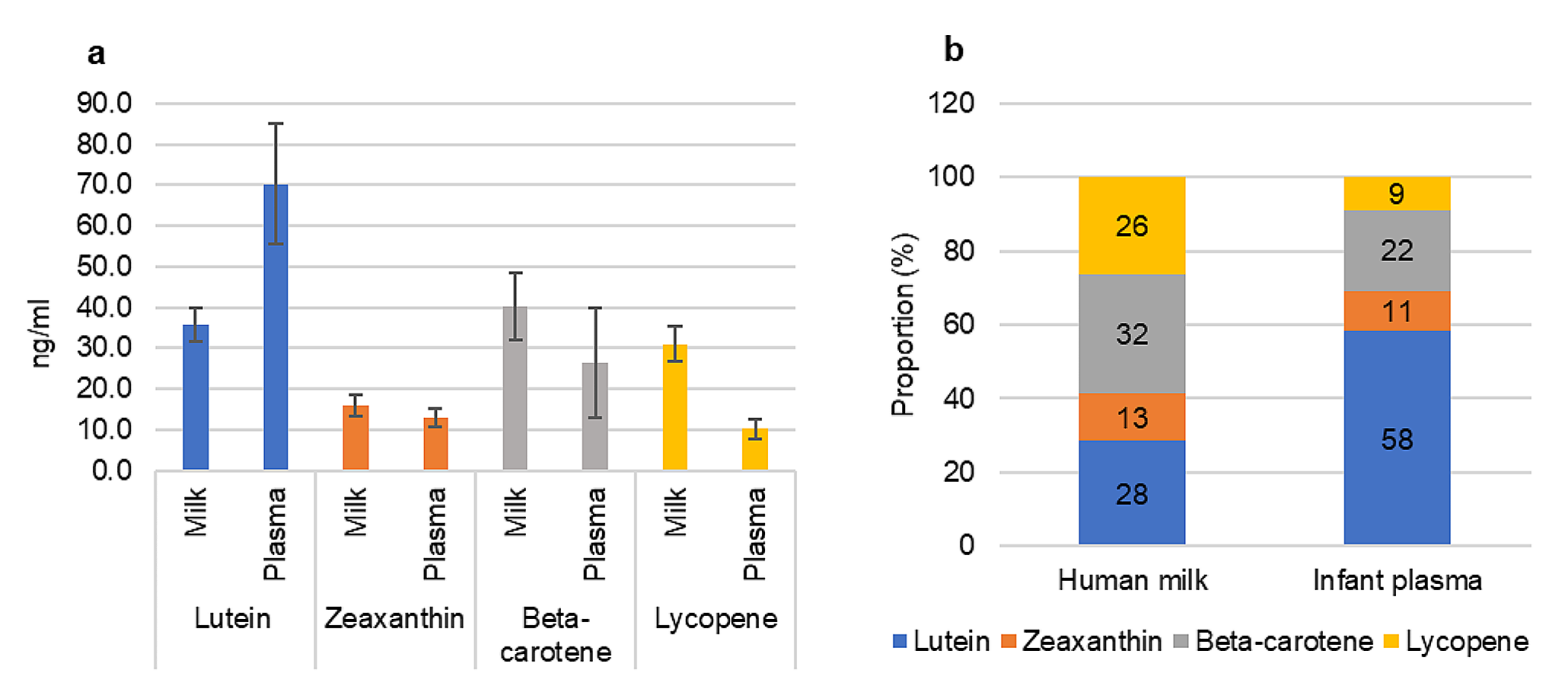
Comparison between carotenoid content of human milk and infant plasma at week 6: **a** Mean (SEM) and **b** Proportions

In infant plasma at week 6 of life, lutein (ng/ml, mean ± SEM) was the predominant carotenoid (76.2 ± 16.6), followed by beta-carotene (24.5 ± 12.8), zeaxanthin (13.6 ± 2.4) and lycopene (11 ± 2.4) as showed in Fig. 4a.

At week 6 post-partum, HM and infant plasma carotenoid distribution differed (Fig. 4a and b). Infant plasma showed lower concentrations of zeaxanthin, beta-carotene and lycopene (Fig. 4a). In HM, lutein accounted for 28% of the total carotenoids, while in infant plasma it accounted for twice as much (58%) (Fig. 4b). No correlation was detected in carotenoid content between HM at week 6 and infant plasma.

Table 2 displays the characteristics of the ROP and non-ROP infants. ROP-diagnosed infants had a significantly lower GA (*p* < 0.001) and BW (*p* < 0.001) than non-ROP infants. Most of the ROP infants were diagnosed after 6 weeks, with the exception of one infant diagnosed at 4 weeks. Length of hospitalization and TPN feeding (median in days) were significantly longer for ROP-diagnosed infants than for non-ROP, (155 versus 51.5, *p* < 0.001 and 27 versus 12.5, *p* = 0.029, respectively). HM fortification was initiated in accordance with our department guidelines, when enteral milk intake reached 80–100 mL/kg/day, and was initiated at a later time (median in days) in ROP-infants than non-ROP infants (13.9 versus 8, *p* = 0.002). Half of the non-ROP infants achieved full feeding (150 mL/kg/day) at day 13 of life while ROP-diagnosed infants needed nearly twice the time (21 days, *p* = 0.016). Finally, transition milk or mature milk did not differ in carotenoid content between mothers of ROP and non-ROP infants.

**Table 1:** Characteristics of study participants

Mothers (*N* = 39)	
Maternal age (years), mean ± SD, (min-max)	32.3 ± 5.4 (20–44)
Parity, *n* (%)	
Primiparous	25 (64)
1	10 (26)
> 1	4 (10)
Pre-pregnancy BMI* (Kg/m^2^), mean ± SD, (min-max)	24.1 ± 4.5 (18.4–32.8)
Pre-eclampsia, *n* (%)	6 (15)
IUGR, *n* (%)	3 (7.7)
Chorioamnionitis, *n* (%)	3 (7.7)
Oligohydramnios, *n* (%)	3 (7.7)
Gestational Diabetes, *n* (%)	3 (7.7)
Cesarean delivery, *n* (%)	27 (69)
Twin delivery, *n* (%)	3 (7.7)
Previous breastfeeding experience, *n* (%)	13 (33)
Vegetarian diet, *n* (%)	4 (10)
Smoking during pregnancy, *n* (%)	3 (7.7)
Smoking while breastfeeding, *n* (%)	2 (5.1)
Smoking exposure during breastfeeding, *n* (%)	7 (18)
Alcohol while breastfeeding, *n* (%)	3 (7.7)
Vitamin supplements during pregnancy, *n* (%)	19 (49)
Vitamin supplements while breastfeeding, *n* (%)	23 (59)
Chronic disease. *n* (%)	5 (13)
**Neonates (** *N* ** = 38)**	
Gestational age (weeks), median (IQR)	29 (27–30)
Birth weight (grams), median (IQR)	1082.5 (940-1306.3)
Gender (female), *n* (%)	20 (53)
5-minute Apgar score, median (IQR)	7.5 (7, 9)
RDS, *n* (%)	31 (82)
BPD, *n* (%)	15 (40)
NEC, *n* (%)	
Stage 2	2 (5.3)
Stage 3	1 (2.6)
IVH, *n* (%)	
Grade 1	10 (26)
Grade 2	2 (5.3)
Sepsis, *n* (%)	5 (13)
ROP**, n (%)	7 (18)
Length of Hospitalization (days), median (IQR)	57.5 (40-90.5)

BMI body mass index, IUGR Intrauterine growth restriction, RDS Respiratory distress syndrome, BPD Bronchopulmonary dysplasia, NEC Necrotizing enterocolitis, IVH Intraventricular hemorrhage, ROP Retinopathy of prematurity SD Standard deviation, IQR Interquartile range, ^*^14 missing, ^**^One missing

**Table 2:** Comparison between ROP-diagnosed infants and non-ROP infants by Mann-Whitney tests

Characteristics	Non-ROP cases (*n* = 30)	ROP cases (*n* = 7)	*p*-value
Gestational age (weeks), median (IQR)	29 (28–30)	24 (24–26)	< 0.001
Birth weight (grams), median (IQR)	1195 (1015–1330)	680 (670–740)	< 0.001
Length of hospital stay (days), median (IQR)	51.5 (37-71.5)	125 (109–146)	< 0.001
Length of TPN feeding (days), median (IQR)	12.5 (9.8–27.5)	27 (18–43)	0.029
Day of life at full feeding (days), median (IQR)	13 (11-16.3)	21 (13–28)	0.016
Start day of Exclusive HM feeding (days), median (IQR)	2 (2-5.3)	2 (1–2)	0.100
Start of Human Milk Fortifier supplementation (days), median (IQR)	8 (6–11)	13.9 (9–20)	0.015
Day of life at diagnosis of ROP (days), median (IQR)	NA	55 (53–73)	NA
Transition milk carotenoids (up to two weeks post-delivery) (ng/ml), median (IQR)	*n* = 23*	*n* = 5**	
Lutein	74.5 (55-106.2)	76.2 (53.9-120.9)	0.771
Zeaxanthin	27 (17.4–37.4)	41.5 (26.4–54.5)	0.264
Beta-carotene	69.8 (34.7-126.6)	81 (44.7-154.6)	0.862
Lycopene	75 (58.2-130.7)	81.5 (71.9-256.6)	0.413
Mature milk carotenoids (above 14 days post-delivery) (ng/ml), median (IQR)	*n* = 27***	*n* = 7	
Lutein	38.6 (26-58.4)	45.1 (38.7–72.7)	0.163
Zeaxanthin	15.6 (10-29.5)	26.1 (18.5–30.4)	0.206
Beta-carotene	38.3 (19.7–59.8)	38.6 (23-81.2)	0.739
Lycopene	33.6 (22-56.5)	28.5 (21.8–49.5)	0.901

ROP Retinopathy of Prematurity, TPN Total parenteral nutrition, NA Not Assessed, IQR Interquartile range, *Five missing, **Two missing, ***One missing

## Discussion

This study is the first report on longitudinal analysis of HM carotenoids in mothers of VLBW and ELBW preterm infants born at 24–34 weeks of gestation.

We showed that lutein, zeaxanthin, beta-carotene, and lycopene concentrations in HM varied from mother-to-mother, in their relative proportion and decreased as lactation progressed up to a plateau at 5 to 6 weeks post-delivery. Lycopene is the most abundant carotenoid in colostrum, followed by beta-carotene and lutein. Similar trends in carotenoid concentrations throughout stages of lactation were reported by Zaidi et al. [3]. The underlying physiological mechanisms for these variations are yet to be elucidated. Zielinska et al. suggested that this unique mechanism of active transport of carotenoids into the mammary gland just after birth may be a maternal evolutionary adaptation to promote infant eye development and reduce inflammation and oxidative stress in neonates [23]. Prior studies have explored the fluctuations and dynamics of human milk, including variations in protein content between preterm and term human milk, as well as variances in other components such as microbiome [24–26].

Differences in carotenoid content between preterm and term HM have been investigated in several studies. Xavier et al. have reported that carotenoid content in colostrum produced by mothers of preterm infants at GA 28–35 weeks, is quantitatively lower than the one of mothers of term infants except for lutein that remained equivalent. However mature milk content of carotenoids was quantitatively and qualitatively similar in both mothers of preterm and term infants [13].

Redeuil et al. evaluated the carotenoid concentrations for post-menstrual age and for post-partum age in term (GA 37–42 weeks) and preterm (GA 28–33 weeks) HM. They found that lycopene was the only carotenoid exhibiting a significantly higher concentration in term than in preterm HM between weeks 1 and 4 post-partum and when compared at equivalent post-menstrual age, preterm milk showed significantly lower levels of beta-carotene, beta-cryptoxanthin, lutein, zeaxanthin, and lycopene compared to their term counterparts [27]. We could not find any reports on the carotenoid content of preterm HM at lower GA than 27–30 weeks. The preterm colostrum in our cohort showed higher carotenoid levels at a similar week postpartum even though their infants were born with a lower GA than those reported by Redeuil (e.g., 156.9 versus 88.6 ng/ml for lutein and 66.9 versus 19.2 ng/ml for zeaxanthin, respectively) [27]. This observation could be attributed to the fact that the Israeli local diet in general resembles the Mediterranean diet built around fruits and vegetables [28].

The lutein and beta-carotene contents of HM in vegetarian mothers were significantly higher than in omnivorous mothers at almost all stages of lactation. These results are in line with other studies that showed that the carotenoid content of HM is correlated to maternal diet [10, 11], lactation stage [2, 29, 30], health status of the mother as well as her alcohol intake and smoking habits [9]. It is reasonable to assume that vegetarian mothers consumed more fruits and vegetables than non-vegetarians. In our study, the content of lutein and zeaxanthin in HM did not correlate to the ones found in infant plasma, in contrast to other publications [2, 31]. There are a number of possible explanations for this observation, one of which could be related to how HM is handled and stored. HM samples collected for the study were stored in a deep freezer and protected from light, in contrast, freshly expressed HM samples were handled following standard NICU protocol and stored at 4 °C, with no protection from light exposure, before infant feeding. It has already been shown that carotenoids, particularly lutein, are light-sensitive, and storage temperatures above deep freezing caused a decrease in concentration [32, 33]. In addition, the bioavailability of carotenoids is affected by several factors such as lipid content of HM and variations in genes associated with carotenoids absorption and metabolism [34]. Further, infant plasma samples contained almost twice as much lutein as HM samples. It has been suggested that milk-fat globules’ specific structure might enhance the absorption of lutein [35]. A recently published review has reported and discussed the preferential selection and accumulation of lutein to specific organs (brain and retina) and that although lutein and zeaxanthin accounted for less than 20% of the total carotenoids in the human diet, their amount in blood plasma increased to about 40% [7].

Additionally, we found no significant difference in HM carotenoid content between mothers of ROP and non-ROP infants. This contrasted with our expectations. Possible reasons for this might be that we only had few cases of ROP-diagnosed infants, not enough to detect any significant differences as well as the multifactorial nature of the pathological process. HM carotenoids play a vital role in an antioxidative network of bioactive HM substances, which provide protective functions against oxidative stress [4]. Prematurity-related conditions such as ROP, necrotizing enterocolitis (NEC) and bronchopulmonary dysplasia (BPD) are associated with damage caused by oxidative stress [36]. Breastfeeding has been linked to lower incidence rates for these severe outcomes [37, 38]. It is yet to be elucidated if maternal supplementation with carotenoids with antioxidative properties could be an effective nutritional therapy against ROP. There are some conflicting results about neonatal lutein supplementation to prevent ROP. It has been reported that term newborn infants who received lutein supplementation in the first days of life had a reduction in free radical formation and oxidative injury [39, 40]. Additionally, Rubin et al. showed that preterm infants fed lutein-supplemented formula seemed to have developed less severe ROP [12]. Others showed trends in reducing the risk of retinopathy, but demonstrated, in a randomized control trial, no significant effect of lutein/zeaxanthin supplementation to preterm infants on threshold ROP, NEC, or BPD, suggesting the need for further research [41]. It is noteworthy that in our study, infants who developed ROP were smaller (in GA and BW) and sicker (needed more oxygen and blood products, more TPN days, achieved full feeding at later time, and lower Apgar score) and solely fed with HM (Human milk fortifier at a later time point). Overall, the amount of HM provided to these infants was lower, therefore their intake of lutein and other carotenoids was lower than in non-ROP infants. None of the ROP infants had a vegetarian mother.

Although the prospective nature of our study is a strength, several limitations need to be mentioned: the small cohort of ROP cases and the fact that exploitable dietary data were not available for the mothers of ROP infants.

## Conclusions

We demonstrated that the carotenoid content of preterm HM is dynamic and shows a high amount of variability between mothers and lactation stages. Carotenoid content decreased as lactation progressed. This study provides useful information on the carotenoid content of HM in mothers of preterm infants. It shows the influence of a vegetable-rich diet on these levels, potentially providing a basis for dietary recommendations to mothers of preterm infants. In addition, fortifying preterm formula with carotenoids has the potential to offer essential developmental benefits, bridging the gap between HM and formula. Further studies are warranted to thoroughly assess the health implications of formula supplementation with carotenoid, especially lutein, for the vulnerable population of preterm infants.

### Electronic supplementary material

Below is the link to the electronic supplementary material.

Supplementary Material 1

Supplementary Material 2

Supplementary Material 3

Supplementary Material 4

## Data Availability

No datasets were generated or analysed during the current study.

## Abbreviations

ANOVA: Analysis of variance
BMI: Body mass index
BPD: Bronchopulmonary dysplasia
BW: Birth weight
ELBW: Extremely low birth weight
GA: Gestational age
GDM: Gestational diabetes mellitus
HM: Human milk
IQR: Interquartile range
IUGR: Intrauterine growth restriction
IVH: Intraventricular Hemorrhage
NEC: Necrotizing Enterocolitis
NICU: Neonatal intensive care unit
RDS: Respiratory Distress Syndrome
ROP: Retinopathy of prematurity
SD: Standard deviations
SEM: Standard error of the mean
TPN: Total parental nutrition
VLBW: Very low birth weight
