# Supplementary material for: Longitudinal analysis of carotenoid content in preterm human milk
Source: Eur J Pediatr. 2024 Mar 21;183(6):2671–82. doi: 10.1007/s00431-024-05485-8 (PMC11098918; doi:10.1007/s00431-024-05485-8)
Supplement: Supplementary file 1 — Supplementary Material 1 [file 431_2024_5485_MOESM1_ESM.pdf]

Supplementary Information:

Longitudinal Analysis of Carotenoid Content in Preterm Human Milk

Adi Uretzky[1,4], Dror Mandel[1,4], Anat Schwartz[1,4], Kira Kaganov[1,4], Daphna Mezad-Koursh[3,4], †Laurence Mangel[1], †Ronit Lubetzky[2.4]

[1]Tel Aviv Medical Center, Department of Neonatology, Dana Dwek Children's Hospital, Tel Aviv 6997801, Israel;

[2]Tel Aviv Medical Center, Department of Pediatrics, Dana Dwek Children's Hospital, Tel Aviv 6997801, Israel

[3]Tel Aviv Medical Center, Division of Ophthalmology, Tel Aviv 6997801, Israel

[4]Faculty of Medicine, Tel Aviv University, Tel Aviv, Israel.

† These authors contributed equally to this work.
* Correspondence: adi.uretzky@gmail.com.

2.4. Laboratory methods

2.4.1. Chemicals and reagents

Chloroform (CHCl3) was purchased from J.T. Baker, USA. Methanol (MeOH), ethanol, Hexane, methyl-tert-butyl ether (MTBE), and tetrahydrofuran (THF) were purchased from Biolab, Jerusalem, Israel. Butylated hydroxytoluene (BHT), Ascorbic acid, and potassium hydroxide (ACS grade, ≥85%) were purchased from Sigma-Aldrich, St. Louis, USA. Unless otherwise specified, all reagents were of HPLC grade or higher. The diluent solution was made as MeOH: CHCl3: MTBE 40:40:20 v/v/v. Individual solvents with 0.1 % BHT (chloroform, ethanol, and hexane) were prepared by adding 1000 mg of BHT into 1L of solvent. Lutein (β, ε-Carotene-3,3′-diol, CAS 127-40-2), Beta-Carotene (β, Beta-Carotene, CAS 7235-40-7), Lycopene (ψ, ψ-Carotene, CAS 502-65-8) and as internal standard (IS), trans-ß-Apo-8'-Carotenal (8′-Apo-beta-caroten-8′-al, CAS 1107-26-2) were purchased from Sigma-Aldrich. Zeaxanthin (β, Beta-carotene-3,3′-diol, CAS 144-68-3) was purchased from Carbosynth.

2.4.2. Carotenoid analysis in HM samples

HM samples were transferred frozen to the laboratory of Frutarom Ldt. where they were further stored frozen at -80°C until analysis. The contents of lutein, beta-carotene, zeaxanthin, and lycopene in HM samples were measured by gradient reversed-phase high-performance liquid chromatography with UV detection (HPLC-UV) by a method modified from Chauveau-Duriot et al. [25].

Stock solutions of standards were prepared by dissolving in Chloroform with 0.1% BHT followed by working calibration solutions in diluent solution MeOH: CHCl3: MTBE 40:40:20 v/v/v with 0.1% BHT in the range 20 to 500 ng/ml (ppb). IS solution was prepared by dissolution of trans-ß-Apo-8'-Carotenal in CHCl3 followed by dilution in diluent solution MeOH: CHCl3: MTBE 40:40:20 v/v/v with 0.1% BHT.

The carotenoids in milk samples were extracted and saponified as briefly described below.

Approximately 1 ml milk was weighed into a 15 ml Falcon tube and 0.5 ml aqueous saturated ascorbic acid solution, 1 ml ethanol with 0.1% (BHT), and 1.5 ml aqueous solution of 50% potassium hydroxide were added. The tube was shaken for 45 min at 400 rpm at 45°C in Incubator 1000 (Heidolph instruments, Schwabach, Germany). The samples were then cooled down on ice, and then mixed vigorously for 30 s with 5 mL of hexane with 0.1% BHT. The tubes were centrifuged at 1300 x g for 2 min at 4°C. The upper organic phase was transferred to a clean 15-mL tube using a glass Pasteur pipette. The liquid/liquid extraction process was repeated, and the organic phases combined. Once completely dried under nitrogen at 30°C, the residue was dissolved in 500 µL of internal standard solution and filtered into injection vials via 0.22µm PTFE filter (DIKMA, ProMax syringe filter,13mm 0.22µm PTFE).

Injections of 20 µl were analyzed on an Agilent modular system with a quaternary pump and fitted with a 250×4.6 ID mm, 5 µm YMC carotenoid column placed in a column oven set at 20°C. The mobile phases were A MeOH, B water, and C MTBE. The eluting gradient program started at 1.0 ml/min at A/B/C 81/4/15 and kept for 10 min, then till 20 min changed to A/B/C 7/3/90 with a flow of 1.4 ml/min. Until 25 min, mobile phase programming changed to starting conditions at A/B/C 81/4/15, and by 26 min flow was reduced to 1.0 ml/m. Detection was performed using UV absorbance detection at 447 nm. Each of the four carotenoids was calculated using a weighted linear regression of analyte to IS area ratio versus analyte to IS amount ratio.

2.4.3. Carotenoid analysis in plasma samples

Carotenoid analysis in plasma samples was performed in the Department of Nutrition-Health and Lipid Biochemistry at the French Institute for Fats and Oils in Canéjan, France (ITERG). The concentration of carotenoid (alpha and beta-carotene, lycopene, lutein, zeaxanthin) in plasma was determined by high-performance liquid chromatography (HPLC, Thermo Scientific, Malboeuf, France) with diode array detector (DAD), according to the modified method of Chauveau-Duriot et al. [25].

Briefly, plasma was deproteinized by the same volume of ethyl alcohol containing 0.02% of BHT, and carotenoids were extracted twice by adding two volumes of n-hexane. The mixture was vortexed for 30 s then centrifuged at 1,000×g for 5 min at 4°C. Both hexane phases were collected and evaporated under nitrogen. The dry residue was dissolved in 200 µl of chloroform/methanol (50/50) with 0.02% of BHT. An aliquot of 10 µl of the sample was injected into an analytical column VYDAC 201TP C18 column (5 µM, 250 × 4,6 mm; VWR, Rosny-sous-Bois, France) and eluted with a gradient of solvent B (Methanol/ethyl acetate (70/30)) from 0% to 100% in 25 min and held at 100% for 15 min at a flow rate of 1 ml/min. The solvent A was water + 0.5 mol/l Ammonium acetate/Methanol (20/80). The absorbance wavelength of the detector was set at 450 nm. The carotenoid concentration was calculated by a linear regression analysis of the peak area versus the concentrations of the carotenoid standard.
