# Supplementary material for: Longitudinal analysis of carotenoid content in preterm human milk
Source: Eur J Pediatr. 2024 Mar 21;183(6):2671–82. doi: 10.1007/s00431-024-05485-8 (PMC11098918; doi:10.1007/s00431-024-05485-8)

Online Resource Fig. S1

Longitudinal Analysis of Carotenoid Content in Preterm Human Milk

Adi Uretzky^1,4^, Dror Mandel^1,4^, Anat Schwartz^1,4^, Kira Kaganov^1,4^, Daphna Mezad- Koursh^3,4^, †Laurence Mangel^1^, †Ronit Lubetzky^2.4^

^1^Tel Aviv Medical Center, Department of Neonatology, Dana Dwek Children’s Hospital, Tel Aviv 6997801, Israel;

^2^Tel Aviv Medical Center, Department of Pediatrics, Dana Dwek Children’s Hospital, Tel Aviv 6997801, Israel

^3^Tel Aviv Medical Center, Division of Ophthalmology, Tel Aviv 6997801, Israel

^4^Faculty of Medicine, Tel Aviv University, Tel Aviv, Israel.

† These authors contributed equally to this work.

*****Correspondence: adi.uretzky@gmail.com.

**Fig. S1** Intra- and Inter-mother variability in carotenoid profile (mean±SEM) of mature milk samples (week 3 to 6) in **a** mothers of ROP diagnosed infants (N=7) and **b** mothers of non-ROP infants (N=30)


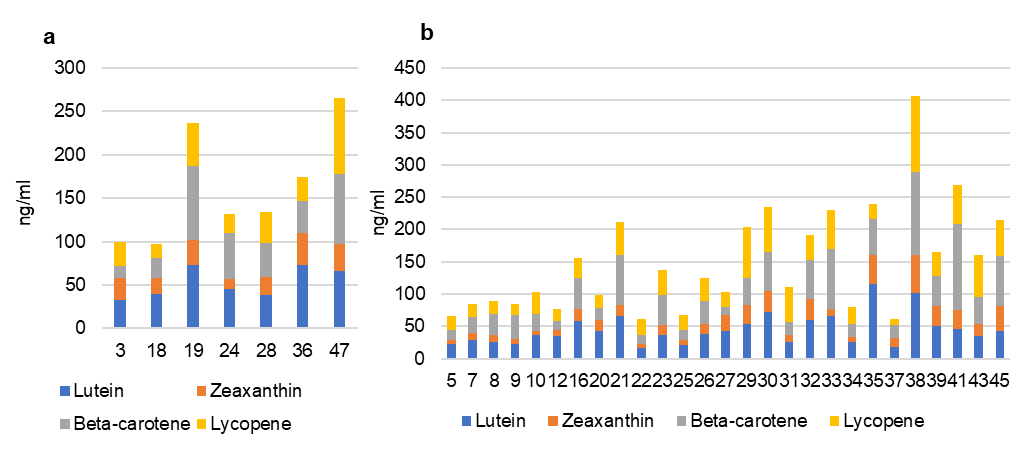

Supplement: Supplementary file 2 — Supplementary Material 2 [file 431_2024_5485_MOESM2_ESM.docx]
