# Supplementary figures and images for: Longitudinal analysis of carotenoid content in preterm human milk
Source: Eur J Pediatr. 2024 Mar 21;183(6):2671–82. doi: 10.1007/s00431-024-05485-8 (PMC11098918; doi:10.1007/s00431-024-05485-8)

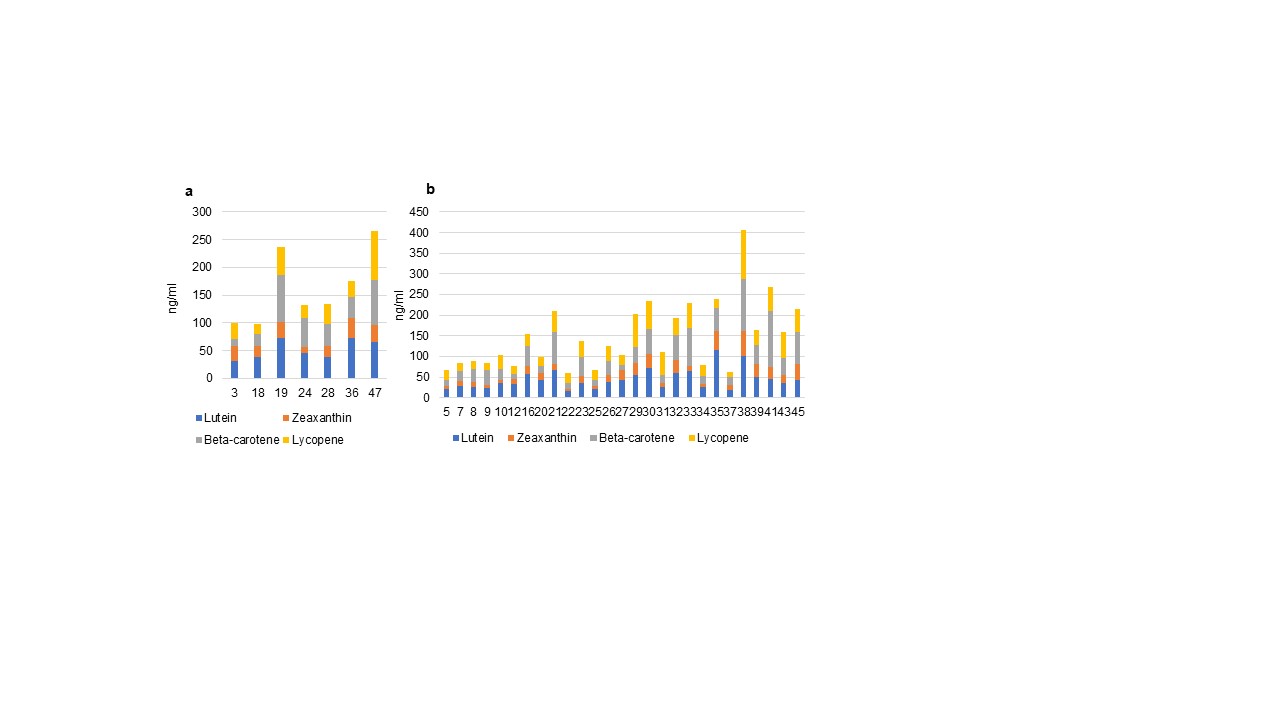

Supplement: Supplementary file 3 — Supplementary Material 3 [file 431_2024_5485_MOESM3_ESM.jpg]
