# Supplementary material for: Longitudinal analysis of carotenoid content in preterm human milk
Source: Eur J Pediatr. 2024 Mar 21;183(6):2671–82. doi: 10.1007/s00431-024-05485-8 (PMC11098918; doi:10.1007/s00431-024-05485-8)
Supplement: Supplementary file 4 — Supplementary Material 4 [file 431_2024_5485_MOESM4_ESM.docx]

Online Resource Table S1

Longitudinal Analysis of Carotenoid Content in Preterm Human Milk

Adi Uretzky^1,4^, Dror Mandel^1,4^, Anat Schwartz^1,4^, Kira Kaganov^1,4^, Daphna Mezad- Koursh^3,4^, †Laurence Mangel^1^, †Ronit Lubetzky^2.4^

^1^Tel Aviv Medical Center, Department of Neonatology, Dana Dwek Children’s Hospital, Tel Aviv 6997801, Israel;

^2^Tel Aviv Medical Center, Department of Pediatrics, Dana Dwek Children’s Hospital, Tel Aviv 6997801, Israel

^3^Tel Aviv Medical Center, Division of Ophthalmology, Tel Aviv 6997801, Israel

^4^Faculty of Medicine, Tel Aviv University, Tel Aviv, Israel.

† These authors contributed equally to this work.

*****Correspondence: adi.uretzky@gmail.com.

**Table S1** Variations in lutein and beta-carotene levels throughout lactation by maternal factors per Mann-Whitney analysis

| Stage of Lactation | Carotenoid (ng/ml) | Vegetarian | Non-vegetarian | p-value | Vaginal delivery | Cesarean delivery | p-value |
| --- | --- | --- | --- | --- | --- | --- | --- |
|  |  | n=4 | n=28 |  | n=10 | n=22 |  |
| Transition* | Lutein | 153.2  (98.4-218.1) | 72.3  (54.1-97.4) | 0.007 | 100.3  (73.5-137.5) | 65.7  (46.0-97.3) | 0.035 |
|  | Beta-carotene | 148.3  (114.6-567.6) | 50.9  (32.6-105.5) | 0.007 | 120.9  (93.5-157.1) | 36.9  (30.4-90.3) | 0.007 |
|  |  | n=4 | n=34 |  | n=12 | n=26 |  |
| Mature** | Lutein | 83.7  (51.0-112.3) | 41.2  (27.6-57.7) | 0.012 | 62.1  (40.4-70.6) | 39.0  (26.2-50.8) | 0.025 |
|  | Beta-carotene | 102.8  (61.1-132.7) | 36.1  (20.0-48.6) | 0.003 | 58.1  (40.3-84.3) | 25.5  (19.45-46.8) | 0.005 |

*Seven missing milk sample, **One missing milk sample
